# Oral Calcidiol Is More Effective Than Cholecalciferol Supplementation to Reach Adequate 25(OH)D Levels in Patients with Autoimmune Diseases Chronically Treated with Low Doses of Glucocorticoids: A “Real-Life” Study

**DOI:** 10.1155/2015/729451

**Published:** 2015-06-01

**Authors:** Miguel Ortego-Jurado, José-Luis Callejas-Rubio, Raquel Ríos-Fernández, Juan González-Moreno, Amanda Rocío González Ramírez, Miguel A. González-Gay, Norberto Ortego-Centeno

**Affiliations:** ^1^Unidad de Medicina Familiar y Comunitaria, Hospital Son Llàtzer, Palma de Mallorca, Spain; ^2^Unidad de Enfermedades Autoinmunes, Hospital Clínico San Cecilio, Granada, Spain; ^3^Servicio de Medicina Interna, Hospital Son Llàtzer, Palma de Mallorca, Spain; ^4^Biomedical Research Institute, ibs. GRANADA, University Hospitals in Granada, University of Granada, Spain; ^5^Servicio de Reumatología, Hospital Marqués de Valdecilla, IDIVAL, Santander, Spain

## Abstract

Glucocorticoids (GCs) are the cornerstone of the therapy in many autoimmune and inflammatory diseases. However, it is well known that their use is a double edged sword, as their beneficial effects are associated almost universally with unwanted effects, as, for example glucocorticoid-induced osteoporosis (GIO). Over the last years, several clinical practice guidelines emphasize the need of preventing bone mass loss and reduce the incidence of fractures associated with GC use. Calcium and vitamin D supplementation, as adjunctive therapy, are included in all the practice guidelines. However, no standard vitamin D dose has been established. Several studies with postmenopausal women show that maintaining the levels above 30–33 ng/mL help improve the response to bisphosphonates. It is unknown if the response is the same in GIO, but in the clinical practice the levels are maintained at around the same values. In this study we demonstrate that patients with autoimmune diseases, undergoing glucocorticoid therapy, often present suboptimal 25(OH)D levels. Patients with higher body mass index and those receiving higher doses of glucocorticoids are at increased risk of having lower levels of 25(OH)D. In these patients, calcidiol supplementations are more effective than cholecalciferol to reach adequate 25(OH)D levels.

## 1. Introduction

Glucocorticoids (GCs) are the cornerstone of the therapy in many autoimmune and inflammatory diseases [1]. However, it is well-known that their use is a double edged sword, as their beneficial effects are associated almost universally with unwanted effects, as, for example, glucocorticoid-induced osteoporosis (GIO), the most widespread type of secondary osteoporosis [2]. Over the last years, several clinical practice guidelines emphasize the need of preventing bone mass loss and reduce the incidence of fractures associated with GC use [3, 4]. Calcium and vitamin D supplementation, as adjunctive therapy, are included in all the practice guidelines, as calcium and vitamin D supplements have been routinely administered in most clinical trials. However, no standard vitamin D dose has been established. One option is to administer a fixed dose ranging between 800 and 1000 IU/day [3], although the best way to achieve the appropriate supply may be the assessment of plasma 25(OH)D levels [4]. Several studies with postmenopausal women show that maintaining the levels above 30–33 ng/mL helps improving the response to bisphosphonates [5–7]. However, it is unknown if the response is the same in GIO [8], but in the clinical practice the levels are maintained at around the same values. In subjects with normal renal function, cholecalciferol, ergocalciferol, or calcidiol can be used to correct vitamin D deficiency. Although calcidiol can be considered more rapid and effective in subjects with vitamin D deficiency [9, 10] to the best of our knowledge, there are no available data on its benefit in patients with GIO.

Taking into account these considerations, we aimed at assessing the factors associated with suboptimal 25(OH)D levels and analyzing if oral calcidiol is more effective than cholecalciferol supplementation to reach adequate 25(OH)D levels in patients with autoimmune diseases chronically treated with low doses of glucocorticoids.

## 2. Methods

### 2.1. Study Design and Patients

This was a real-life study in which patients with autoimmune diseases, undergoing GC therapy, followed up at the Outpatient Clinic of the Systemic Autoimmune Diseases Unit at San Cecilio Hospital in Granada (Spain) (a university teaching hospital), between January 2010 and September 2013 were assessed. The study was approved by the Clinical Research Ethics Committee of the San Cecilio Hospital. All participants gave their written consent in accordance with the Declaration of Helsinki.

### 2.2. Inclusion Criteria

The inclusion criteria included patients 18 years old and older who have been on GC therapy, for more than three months before the date of recruitment, and who were expected to continue receiving glucocorticoids in a stable dose, because of the underlying autoimmune disease, for at least another three months, and in whom their doctor had indicated the use of calcidiol or cholecalciferol for the prevention of GIO.

### 2.3. Exclusion Criteria

The exclusion criteria included patients who had been treated with 6-methylprednisolone pulses or who required higher doses of prednisone 15 mg/d for disease control, and those suffering from other diseases such as neoplasia, liver disease, hyperparathyroidism, hypercalcemia, hypercalciuria, hypophosphatemia, or renal insufficiency (creatinine levels >1.5 mg/dL) were excluded.

### 2.4. Procedure

At the time of inclusion relevant clinical data including the dose of GC and the drugs used to avoid the potential bone mass loss mediated by GC, including type of vitamin D supplement (cholecalciferol or calcidiol), was assessed. At each visit, clinical data related to the patient's disease, as well as his/her adherence to the indicated treatment, were recorded. The interval between two visits was not longer than 6 months with a follow-up of one year. The average prednisone dose was calculated by dividing the accumulated prednisone dose between the days of follow-up. Laboratory determinations in fasting conditions were done at inclusion. During follow-up, 25(OH)D levels were also measured. At least two determinations were obtained for each patient, one during spring-summer (from April 1 to September 30) and another during fall-winter (from October 1 to March 31). We established the mean 25(OH)D level for each patient considering the values obtained in both periods. Regarding vitamin D, values ≥30 ng/mL were considered optimal and values <30 ng/mL were considered suboptimal (insufficient at concentrations between 15 ng/mL and 30 ng/mL and deficient at concentration <15 ng/mL).

During the study visits, patients were asked for any adverse events. Serum calcium levels and urinary calcium excretion (calcium/creatinine ratio in spot urine) were assessed at each visit.

### 2.5. Measurements of 25(OH)D

25(OH)D assessment was done using the IDS-iSYS25OHD kit supplied by Immunodiagnostic Systems Ltd (Boldon, England), a chemiluminescence direct competitive immunoassay for quantitative determination of total serum or plasma25(OH)D. The intra- and interassay coefficient of variations (VC%) were below 12.1% and 16.9%, respectively.

## 3. Statistical Analysis

For the analysis of the data the Kolmogorov-Smirnoff test was applied to establish the goodness of fit to normality for the variables studied. Descriptive statistics (mean ± SD) were determined for all variables. Student's *t*-test was used to compare quantitative variables. Categorical variables and proportions were analysed using the Chi-square test. Intergroup comparison of 25(OH)D concentrations was done with one-way ANOVA. Correlation analyses were performed with Pearson's or Spearman's rank order correlation coefficients, where appropriate. Finally, a logistic regression analysis was performed to assess the factors that could independently influence the presence of suboptimal levels of 25(OH)D. The strength of the association was expressed by odds ratio (OR) with confidence interval of 95%. In all analyses, *p* value <0.05 was interpreted as statistically significant. All statistical analyses were performed using SPSS version 17.0 (IBM, Armonk, NY, USA).

## 4. Results

One hundred forty-seven subjects (all Caucasian) were included in the analysis (67 lupus erythematosus; 35 systemic vasculitis; 11 rheumatoid arthritis; 10 sarcoidosis; 9 inflammatory myopathies; 15 others). All the patients were from Granada, a sunny Andalucian city (37° 11′′ north latitude) with 3016 hours of sun per year. Eighty-six patients received cholecalciferol (800 IU/day) plus 3000 mg/day of calcium carbonate, in two doses, and 49 patients calcidiol (10.640 IU/4 weeks [equivalent 354 IU/d]). At the time of inclusion in the study, the patients were already on calcidiol or cholecalciferol for at least three months. Patients treated with calcidiol were instructed to make a diet with at least 1200 mg/d of calcium. Twelve patients, who were prescribed treatment, received no supplements by patient personal choice, fearing possible adverse effects. The mean prednisone or equivalent dose was 5.24 ± 2.91 mg/d. Basal characteristics of the patients included in the study are shown in Tables 1 and 2. Patients treated with calcidiol had a lower BMI (28.9 ± 5.8 versus 26.4 ± 4.5; *p* = 0.010); and women in this group were less frequently menopausal (46.5 versus 67.7; *p* = 0.028).

Assessment of the mean annual 25(OH)D levels in the whole group of 147 patients revealed normal, insufficient, and deficient levels in 46.9%, 40.8%, and 12.2% of the patients, respectively. During the spring-summer period, 55.1% of the 147 patients had 25(OH)D levels >30 ng/mL, 34.7% had insufficient levels, and 10.2% had deficient levels. During the fall-winter period, 42.3% of them had normal 25(OH)D levels, 46.2% had insufficient levels, and 11.5% deficient levels.  Table 3 shows the levels of 25(OH)D in the total sample and in patients treated with cholecalciferol or calcidiol.

A statistically significant negative correlation between 25(OH)D levels and body mass index (BMI) (*r* = −0.19; *p* = 0.03) or daily prednisone dose (*r* = −0.25; *p* = 0.003) was observed.

**Figure 1:**
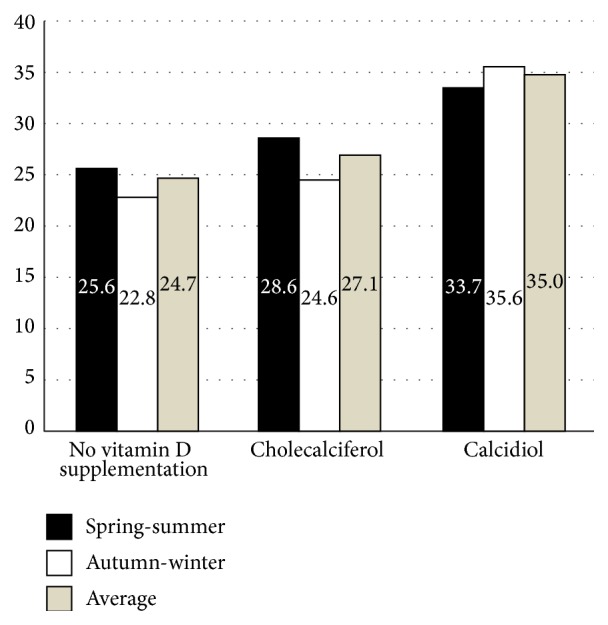
25(OH)D levels (ng/mL) throughout the year in patients receiving cholecalciferol or calcidiol or not taking vitamin D (controls).

With regard to the season of the year and vitamin D intake, we found that patients treated with calcidiol had higher 25(OH)D concentration when compared with those treated with cholecalciferol or with those who did not receive vitamin D3 supplements; however, these differences were only statistically significant in winter (*p* < 0.0001) or when 25(OH)D annual mean value was assessed (*p* = 0.002) (Figure 1).

All variables of interest were tested for association with suboptimal 25(OH)D levels. The results are presents in Table 4. The correct classification rate of the model was 65%, with a specificity of 55.7% and a sensitivity of 73%. Daily prednisone dose (mg/d) was independently associated with suboptimal 25(OH)D levels (OR = 1.83; CI 95%, 1.021–1.372; *p* = 0.026) and calcidiol supplements were a protective factor for suboptimal 25(OH)D (OR = 0.441; CI 95% 0.209–0.928; *p* = 0.031).

No hypercalcemia or hypercalciuria was observed in any patient, regardless of treatment.

## 5. Discussion

There is still controversy over the 25(OH)D levels that should be considered as normal in humans. The 25(OH)D concentration in people living like our ancestors did, such as the Masai, and in children and adults from South Africa and Gambia ranges between 30 and 45 ng/mL. In this regard, some authors concluded that these ranges are the desirable values for optimal skeletal and extraskeletal health [11], while others consider a level above 20 ng/mL as the appropriate concentration [12]. In our study we defined >30 ng/mL as the adequate concentration for 25(OH)D. This was based on different studies that support the claim that this concentration is optimal to improve the response to the treatment with bisphosphonates in postmenopausal women with osteoporosis [5–7, 13], because some authors believe that the beneficial extraskeletal effects mediated by vitamin D are achieved when higher 25(OH)D concentration is reached [14]. Thus, considering 30 ng/mL as the cutoff concentration, the first result unveiled from our study was the high prevalence of suboptimal 25(OH)D levels, despite the fact that most patients were taking oral vitamin supplements. This high prevalence of vitamin insufficiency has been consistently observed in different countries, in both healthy people and individuals with different diseases [15], particularly in those with autoimmune and inflammatory diseases [16, 17]. According to our results, a fixed supply of vitamin D is not always sufficient to maintain 25(OH)D concentrations above 30 ng/mL.

We found a correlation between BMI and 25(OH)D levels. In keeping with our findings, a recent meta-analysis disclosed a significant inverse weak correlation between serum 25(OH)D levels and BMI in population of adults [18]. The only exception was found in women living in developing countries [18]. This finding supports the need of providing higher vitamin D doses to subjects with high BMI.

In our study, a significant negative correlation between 25(OH)D levels and the daily dose of prednisone was found. Although some authors have found normal 25(OH)D levels in patients receiving GC [19, 20], a recent meta-analysis, in which patients taking at least 400 IU/day (10 *μ*g/day) of vitamin D were excluded, disclosed suboptimal 25(OH)D levels in subjects treated with GC [21]. This was also the case in the National and Nutrition Examination Survey (NHANES) [22]. GCs increase the catabolism of 25(OH)D but they may also induce weight gain, which, in turns, is associated with lower 25(OH)D levels. There are many other negative effects of GC on 25(OH)D levels. In this regard, dexamethasone increases renal expression of vitamin D-24-hydroxylase and the expression of 24-hydroxylase mRNA [23, 24], which degrades vitamin D metabolites such as 25(OH)D and 1,25(OH)_2_D. A functional cooperation between the GC receptor, C/EBP*β*, and the vitamin D receptor (VDR), which increases 24-hydroxylase transcription, has also been described [25]. In addition, the administration of prednisolone in rats inhibits the activity of vitamin D3 25-hydroxylase [26]. These effects may help to explain the low 25(OH)D concentration found in patients of our study that received cholecalciferol. These negative effects are good reasons to determine 25(OH)D levels and probably the administration of higher vitamin D doses, preferably as calcidiol, in GC treated patients to prevent the combined negative effects on bone mediated by GC and hypovitaminosis D [21]. Some trials have also demonstrated an advantage of calcidiol in augmenting BMD in patients after kidney or heart transplantation, most probably bypassing the inhibition of 25 hydroxylases by chronic corticosteroid therapy [27, 28].

Although the number of GC-treated patients who did not receive any type of vitamin D supplementation in our series was small, it is important to highlight that three-quarters of them had suboptimal 25(OH)D levels (75%). This prevalence was reduced in 16.9% and 36.2% in patients treated with cholecalciferol or calcidiol, respectively, despite the fact that patients receiving calcidiol received an average daily dose of vitamin D less than those who received cholecalciferol (354 versus 800 IU/d). In the multiple logistic regression analysis, only the daily dose of prednisone and the calcidiol supplements help to explain suboptimal 25(OH)D levels. These findings are in agreement with data from the general population. Bischoff-Ferrari et al. in a randomized study compared the efficacy of calcidiol versus cholecalciferol in the attainment of the desired serum 25(OH)D levels in healthy postmenopausal women with vitamin D deficiency. They showed that serum 25(OH)D concentration was twofold increased after just 2 weeks using a daily dose of 20 *μ*g of calcidiol reaching 30 ng/mL and then going to 69.5 ng/mL at the end of the intervention. Conversely, the rise in serum 25(OH)D levels determined by cholecalciferol was slow, plateauing at 31 ng/mL after 11 weeks [29]. Cashman et al. determined that each microgram of calcidiol was five times more effective to raise serum 25(OH)D in older adults during winter in comparison to cholecalciferol [30], and Jetter et al. determined that calcidiol given daily, weekly, or as a single bolus is about 2-3 times more potent in increasing plasma 25(OH)D concentrations than vitamin D3 in healthy females, thus allowing to get concentrations of 30 ng/mL more rapidly and reliably than with vitamin D3 [10].

As shown by Stamp, the administration of a single oral dose of calcidiol produced a more rapid and significant increase in serum 25(OH)D concentration compared to a single oral dose of cholecalciferol, which produced a very slow increase of serum 25(OH)D levels because of the intermediate hepatic 25-hydroxylation [31]. This supported an advantage of calcifediol over cholecalciferol in correcting vitamin D deficiency. Indeed, calcidiol is more soluble in organic solvents. This property influences its intestinal absorption, its protein transport in the blood, and the whole body distribution. The greater affinity for protein binding may make 25(OH)D more available for internalization in tissues devoted to the control of mineral homeostasis such as the kidneys and the parathyroids expressing the megalin-cubilin system of endocytic receptors [32].

The main limitation of our study is that it was not a clinical trial but a real-life study, and we do not have levels of 25(OH)D before initiating vitamin D supplement. Patients received calcidiol or cholecalciferol following medical indications and not in a random manner, which could lead to intergroup differences. Nevertheless, it is well-known that, in some cases, the efficacy of an interventional study is better portrayed with real-life studies [33]. We feel that the fact that in our study patients had been included consecutively may contribute to displaying the actual population of patients treated with GC.

An additional limitation of our study was that we just conducted a dietary survey as far as calcium intake is considered, not regarding the possible intake of vitamin D, although it must be emphasized that the rate of vitamin D supplementation in food in Spain is very low [34], and all of the patients live in the same Spanish province.

In summary, despite vitamin D supplementation, there is a high prevalence of suboptimal 25(OH)D concentration in patients treated with low doses of GC, for the management of different autoimmune diseases. Patients with higher BMI and those that receive higher doses of GC are at greater risk of having insufficient levels of 25(OH)D and require greater surveillance. Calcidiol intake contributes to lower risk of suboptimal 25(OH)D levels than cholecalciferol and may constitute an affordable source of vitamin D to prevent GIO.

## Figures and Tables

**Table 1:** Baseline characteristics of the subjects included in the study.

	*N*	Mean	SD	%
Age	147	56.06	16.11	
Gender				
Men	30			20.4%
Female				
Menopause				
No	49			33.3%
Yes	68			46.3%
Weight (kg)	147	72	16	
Height (cm)	147	160	9	
BMI (kg/m^2^)	147	27.91	5.47	
Discontinuation of glucocorticoid therapy				
Yes	14			9.5%
No	133			90.5%
Prednisone (mg/d)	147	5.24	2.91	
Vitamin D				
No supplements (refused)	12			8.2%
Cholecalciferol (vitamin D3)	86			58.5%
Calcidiol (25[OH]D3)	49			33.3%

**Table 2:** Baseline characteristics of the cholecalciferol and calcidiol groups.

	Cholecalciferol	Calcidiol	*p*

Age (mean ± SD)	56.6 ± 14.5	57.4 ± 19.0	NS
Gender (*N*, %)			
Men	21 (24.4)	6 (12.2)	NS
Female	65 (75.6)	43 (87.8)	
Menopause (*N*, %)	44 (67.7)	20 (46.5)	0.028
BMI (mean ± SD)	28.9 ± 5.84	26.40 ± 4.53	0.010
Prednisone, mg/day (mean ± SD)	5.2 ± 2.74	4.0 ± 2.59	NS

**(a):** 

				*N* = 147	%
25(OH)D	Optimal		≥30 ng/mL	81	55.1%
Spring-summer	Suboptimal	Insufficient	15–30 ng/mL	51	34.7%
		Deficient	<15 ng/mL	15	10.2%

25(OH)D	Optimal		≥30 ng/mL	55	42.3%
Autumn-winter	Suboptimal	Insufficient	15–30 ng/mL	60	46.2%
		Deficient	<15 ng/mL	15	11.5%

25(OH)D	Optimal		≥30 ng/mL	69	46.9%
Average per year	Suboptimal	Insufficient	15–30 ng/mL	60	40.8%
		Deficient	<15 ng/mL	18	12.2%

**(b):** 

				*N* = 86	%
25(OH)D	Optimal		≥30 ng/mL	44	51.2%
Spring-summer	Suboptimal	Insufficient	15–30 ng/mL	34	39.5%
		Deficient	<15 ng/mL	8	9.3%

25(OH)D	Optimal		≥30 ng/mL	26	35.6%
Autumn-winter	Suboptimal	Insufficient	15–30 ng/mL	36	49.3%
		Deficient	<15 ng/mL	11	15.1%

25(OH)D	Optimal		≥30 ng/mL	36	41.9%
Average per year	Suboptimal	Insufficient	15–30 ng/mL	35	40.7%
		Deficient	<15 ng/mL	15	17.4%

**(c):** 

				*N* = 49	%
25(OH)D	Optimal		≥30 ng/mL	33	67.3%
Spring-summer	Suboptimal	Insufficient	15–30 ng/mL	11	22.4%
		Deficient	<15 ng/mL	5	10.2%

25(OH)D	Optimal		≥30 ng/mL	27	58.7%
Autumn-winter	Suboptimal	Insufficient	15–30 ng/mL	17	37.0%
		Deficient	<15 ng/mL	2	4.3%

25(OH)D	Optimal		≥30 ng/mL	30	61.2%
Average per year	Suboptimal	Insufficient	15–30 ng/mL	17	34.7%
		Deficient	<15 ng/mL	2	4.1%

**Table 4:** Factors linked to suboptimal 25(OH)D levels in the univariate and multiple logistic regression model, in the average of annual determinations.

Factor	Univariate logistic regression analysis		Multiple logistic regression analysis	
	*p* value	OR (95% CI)	*p* value	OR (95% CI)
Age	0.88	1.00 (0.98–1.02)	NS	
BMI	0.32	1.03 (0.97–1.10)	NS	
Prednisone dosage (mg/d)	0.011	1.230 (1.04–1.39)	0.03	1.20 (1.04–1.39)
Calcidiol versus cholecalciferol	0.027	0.439 (0.21–0.91)	0.03	0.44 (0.21–0.93)
